# Commentary to “Dosing vitamin C in critically ill patients with special attention to renal replacement therapy: a narrative review”

**DOI:** 10.1186/s13613-020-00710-7

**Published:** 2020-07-06

**Authors:** J. M. Morán, I. Herrera-Peco

**Affiliations:** 1 Nursing and Occupational Therapy College, Nursing Department, University of Extremadura, Cáceres, Spain; 2 Faculty of Health Sciences, Alfonso X El Sabio University, Villanueva de la Cañada, Madrid, Spain

## Main text

We have read with interest the manuscript from Honore et al. in *Annals of Intensive Care* about the dose adjustments of vitamin C in critically patients undergoing renal replacement therapy [1]. After a detailed review, we would like to highlight an inaccurate literature review performed in this study that may raise uncertainty in the conclusions of Honore and colleagues.

The authors included in their study the Wu et al.’s case report of a patient with hemolytic jaundice induced by pharmacological dose of ascorbic acid in glucose-6-phosphate dehydrogenase (G6PD) deficiency [2]. This manuscript is used in Honore et al. narrative review discussion to support an important conclusion related to the dose of up to 6 g/day (vitamin C) is not contraindicated in patients with G6PD deficiency [2]. However, the study from Wu et al. was retracted from *Medicine (Baltimore)* journal in 2019 (online: 2019 November 27) [3], because the accuracy or validity of the results is questionable and does not support the hypothesis, making the Wu et al.’s manuscript factually incorrect and inadequate to support Honore et al.’s previous conclusion.

The authors searched for studies and used studies published in 2020, like Fujii et al.’s study [4], so the retraction note, published online in 2019 November [3], should be observed.

We conclude that the exclusion of Wu et al.’s study does not probably affect the overall conclusions of Honore et al.’s study, but it affects directly the particular conclusion exposed previously because Wu et al.’s study failed to provide valid scientific evidence.

We recommend to exclude it, but a good and accurate literature review is a key element in high-quality original studies.

## Data Availability

Not applicable.

## Abbreviation

G6PD: Glucose-6-phosphate dehydrogenase
